# Effect of chest compressions in addition to extracorporeal life support on carotid flow in an experimental model of refractory cardiac arrest in pigs

**DOI:** 10.1016/j.resplu.2024.100826

**Published:** 2024-11-09

**Authors:** Sergey Gurevich, Rajat Kalra, Marinos Kosmopoulos, Alexandra M Marquez, Deborah Jaeger, Mitchell Bemenderfer, Danielle Burroughs, Jason A Bartos, Demetris Yannopoulos, Sebastian Voicu

**Affiliations:** a Division of Cardiology, Department of Medicine, University of Minnesota School of Medicine, Minneapolis, MN, United States; b Center for Resuscitation Medicine, University of Minnesota School of Medicine, Minneapolis, MN 55401, United States; c Division of Pediatric Critical Care Medicine, Department of Pediatrics, University of Minnesota School of Medicine, Minneapolis, MN, United States; d INSERM U 1116, University of Lorraine, Vandœuvre-lès-Nancy, France; e Assistance Publique Hôpitaux de Paris, Hôpital Lariboisière, Université Paris Cité, INSERM UMR-S 1144, France

**Keywords:** Cerebral perfusion, Cardiopulmonary resuscitation, Hemodynamic optimization

## Abstract

**Background:**

Extracorporeal life support (ECLS) provides organ perfusion in refractory cardiac arrest but during the initiation of ECLS mean arterial pressure (MAP) and carotid flow may be suboptimal due to hypotension and/or insufficient flow. We hypothesized that cardiopulmonary resuscitation (CPR) in addition to ECLS may increase carotid flow and MAP compared to ECLS alone.

**Methods:**

Observational pilot study comparing hemodynamic parameters before and after CPR cessation in pigs supported by ECLS for experimental refractory cardiac arrest. Pigs were anesthetized, ventricular fibrillation was induced for 3 min, automated CPR performed for 30 min, ECLS was initiated then CPR stopped.

Variables averaged over 3 s were compared between the last 3 s of CPR + ECLS and 3, 6, 30 s, and 5 and 10 min of ECLS alone. Data are expressed as medians (25–75 interquartile range) and compared using paired samples Wilcoxon test.

**Results:**

Nine pigs were included, ECLS was initiated at 2.7 (2.3–2.8) L/min. MAP during CPR + ECLS was 56(53.0–59.2) mmHg, *versus* 50(45–57)mmHg, 52(46–59)mmHg, 61(50–63)mmHg, 57 (54–66)mmHg, 54 (47–58)mmHg of ECLS alone, p = 0.50, 0.61, 0.70, 0.44, 0.73 respectively. Carotid flow was 113(78–119) ml/min during CPR + ECLS *versus* 99(79–110)ml/min, 100(81–110)ml/min, 96(60–122)ml/min, 118 (101–130)ml/min, 124 (110–141)ml/min, p = 0.41, 0.52, 0.73, 0.33, 0.20 respectively. When ECLS was initiated at lower flow, 1.5 L/min (one pig), MAP decreased from 59 to 45 mmHg, and carotid flow from 78.2 to 32.5 ml/min after 3 s of ECLS alone.

**Conclusion:**

Stopping CPR after effective ECLS initiation does not decrease MAP or carotid flow. Future studies may evaluate augmenting low flow ECLS with CPR.

## Highlights

• Stopping CPR after effective ECLS initiation does not decrease mean arterial pressure.
• Stopping CPR after effective ECLS initiation does not decrease carotid flow.
• Stopping CPR after effective ECLS initiation decreases central venous pressure.

## Graphical abstract

**Figure:**
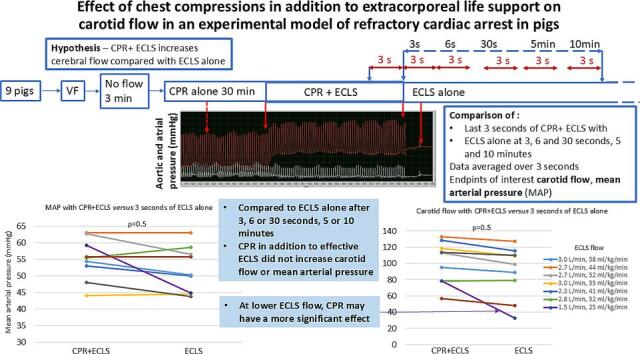

## Introduction

During refractory cardiac arrest, the main initial cardiovascular support is cardiopulmonary resuscitation (CPR)^1^ followed by extracorporeal life support (ECLS) if it is indicated and available.^2^ Once ECLS is initiated, CPR is stopped and hemodynamic status including arterial pressure and blood flow is optimized to achieve optimal cerebral and systemic organ perfusion.^3^, ^4^, ^5^ Optimizing arterial pressure requires vasopressor infusion and invasive arterial pressure monitoring, and from the initiation of ECLS until the invasive arterial pressure monitoring becomes operational, some patients may have low blood pressure and low carotid flow. Continuing CPR during this period may have an effect of increased arterial pressure and carotid flow through additional flow generated by the chest compressions.

In some therapeutic strategies, CPR was evaluated as a complement to circulatory assist devices that do not provide full support, like axial pumps^6^ and was performed to ensure organ perfusion in regional perfusion strategies supplying most of the assist device output to the brain.^7^.

Recent guidelines^8^ suggest that full ECLS support may be achieved with 3–4 L/min flow, but if this optimum support is not initially achieved, pursuing CPR in addition to the ECLS may improve pressure and flow until the ECLS flow is optimal.

We therefore performed an observational pilot study hypothesizing that CPR in addition to ECLS may generate higher MAP and carotid flow, compared to the ECLS support alone, in an experimental model of refractory cardiac arrest model in pigs.

## Material and methods

### Design of the study

This was an *a posteriori* observational ancillary pilot study using data from an experimental randomized study in pigs, performed in the Advanced Preclinical Imaging Center of the University of Minnesota, Minneapolis, Minnesota, USA.

The study received approval from the University of Minnesota Institutional Animal Care and Use Committee (protocol ID: 2212-40647A). The protocol took place under the supervision of a certified, licensed veterinarian who ensured all the procedures took place in compliance with the ARRIVE guidelines (Animal Research: Reporting In Vivo Experiments).^9^.

**Fig. 1:**
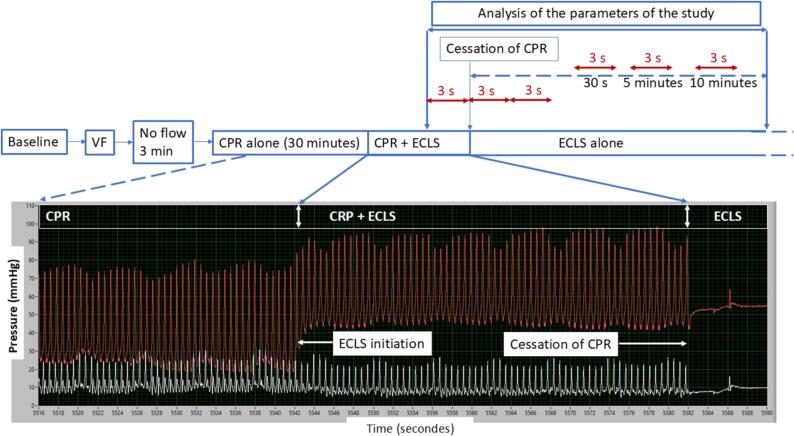
Outline of the study showing the initiation of the ECLS and CPR cessation. VF – ventricular fibrillation, CPR – cardiopulmonary resuscitation, ECLS – extracorporeal life support. Dashed lines represent events extending beyond the time range of the graph. Time intervals of 3 s depicted in red represent the interval over which parameters were averaged. The white curve represents right atrial pressure, and the red curve the aortic pressure. (For interpretation of the references to color in this figure legend, the reader is referred to the web version of this article.). (For interpretation of the references to color in this figure legend, the reader is referred to the web version of this article.)

**Fig. 2:**
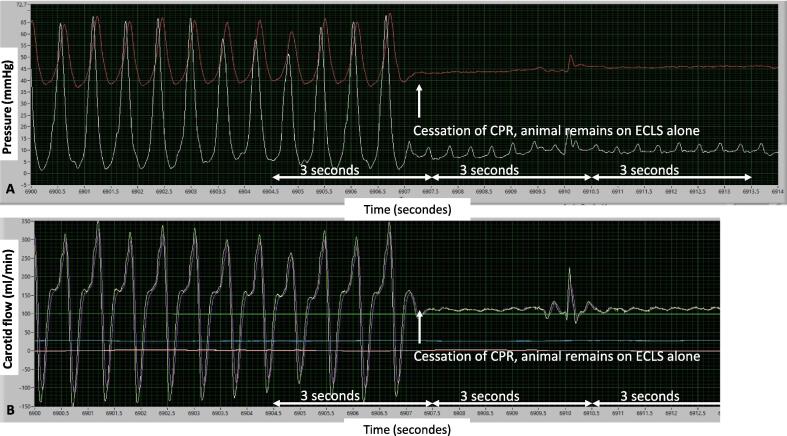
Example of analysis of the hemodynamic parameters of the study before and after CPR cessation. Panel A shows right atrial pressure (white curve) and aortic pressure (red curve). Panel B shows carotid flow. CPR – cardiopulmonary resuscitation, ECLS – extracorporeal life support Time intervals of 3 s over which parameters were averaged are shown for the CPR + ECLS, 3 s of ECLS alone and 6 s of ECLS alone in one of the pigs. (For interpretation of the references to color in this figure legend, the reader is referred to the web version of this article.). (For interpretation of the references to color in this figure legend, the reader is referred to the web version of this article.)

This study compared parameters during the treatment with CPR and ECLS (CPR + ECLS), with parameters immediately after cessation of CPR, when the pigs were supported by ECLS alone, the pigs being their own controls (Fig. 1, Fig. 2).

The objective of the study was to compare the two endpoints of interest, carotid flow and MAP during the last 3 s of CPR + ECLS with the carotid flow and MAP during ECLS alone at 3 s, 6 s, 30 s, and 5 and 10 min after stopping CPR.

### Experimental model of cardiac arrest

The protocol for sedation, mechanical ventilation, monitoring and ECLS initiation was previously described.^10^, ^11^ Female pigs were sedated using intramuscular ketamine (1,000 mg) intubated and sedation was pursued with inhaled isoflurane at a dose of 0.8 % to 1.2 % during mechanical ventilation.

We placed a 6F sheath in the left femoral artery, used for the insertion of a Millar catheter (Mikro-Tip Transducer, Millar Instruments, Houston, Texas) to monitor the arterial pressure.

We exposed the left carotid with a cut down and we placed an ultrasound flow probe to monitor carotid blood flow (Transonic 420 series multichannel, Transonic Systems, Ithaca, NY). A 7F sheath was placed into the left internal jugular vein to administer medication and to accommodate a Millar catheter advanced into the right atrium to monitor the central venous pressure.

We inserted 6F sheaths into the right femoral artery and vein which were subsequently used for cannulation, which included dilation and placement of a 21F venous cannula and 15F arterial cannula at 30 min for systemic ECLS initiation.

Intracranial pressure (ICP) was measured by a Millar catheter inserted in the left lateral cerebral ventricle through a burr hole performed in the skull.

The esophageal temperature was continuously monitored and was maintained at 34 °C using air blankets (Bair Hugger, Augustine Medical, Eden Prairie, MN, USA). Intravenous saline 0.9 % was used for fluid repletion and was administered at a rate of 3 ml/kg/hour after intubation, until ventricular fibrillation was induced. No fluid repletion was administered during cardiac arrest until the ECLS was initiated. After ECLS initiation, fluid repletion at the same rate was restarted and fluid boluses could be administered if the animals were hypovolemic especially if chugging/chatter of the ECLS tubing was observed.

Pigs received heparin 100 IU/kg after vascular access was established. Ventricular fibrillation was induced by a pacing wire advanced in the right ventricle through the right internal jugular vein sheath and by stimulating the right ventricle at 300 pulses/minute.^11^.

As shown in Fig. 1, ventricular fibrillation was left untreated for 3 min corresponding the no-flow period of human cardiac arrest, then CPR was initiated using mechanical chest compressions with a custom-made device, at a frequency of 100 compressions per minute with 4.5 cm compression depth and a duty cycle of 50 % compression.^10^ During CPR, pigs were ventilated with room air with a respiratory rate of 10/min, and a tidal volume of 10 ml/kg. After 10 min of basic life support, advanced life support (ALS) was started and 0.5 mg of epinephrine was administered every 5 min together with 20 mEq of bicarbonate at 10 min and 20 min of CPR.

ECLS was initiated after 20 min of ALS corresponding to 30 min of CPR (Fig. 1) with a CARDIOHELP® device (Maquet Cardiovascular, Wayne, New Jersey). The level of the initial ECLS flow was not pre-specified in the protocol but the targeted ECLS flow was 2.5–3 L/min. The research team could start the ECLS at 2.5–3 L/min or progressively increase flow over until 2.5–3 L/min was achieved. After initiation of ECLS, we targeted a MAP > 65 according to post-resuscitation care guidelines.^5^.

### Parameters analyzed in the study

We included pigs which had at least 3 s of treatment with CPR + ECLS after the initiation of the ECLS. The duration of 3 s was chosen arbitrarily to allow for the pressures and flow to arrive to a steady state after the initiation of the ECLS.

We excluded pigs who did not have a treatment with CPR + ECLS (ECLS was initiated after CPR cessation) or had a progressively increasing ECLS flow as shown by the recorded flows on the ECLS device, because this study evaluates hemodynamic parameters in animals in which ECLS was initiated at constant as opposed to progressively increasing ECLS flow.

Hemodynamic parameters and ICP during the treatment with CPR + ECLS were compared with parameters after 3, 6, 30 s, 5 and 10 min of the treatment with ECLS alone. Parameters were averaged over 3 s.

### Statistical analysis

Numerical parameters were expressed as medians (interquartile 25–75) and compared with paired Wilcoxon test, animals being their own controls. Categorical variables were expressed as numbers (percentages).

Variables were compared between the CPR + ECLS treatment and the following points: baseline before VF, end of CPR alone, and the endpoints of the study 3, 6, 30 s, 5 and 10 min of ECLS alone. A p-value < 0.5 was considered significant.

## Results

### Included animals

**Fig. 3:**
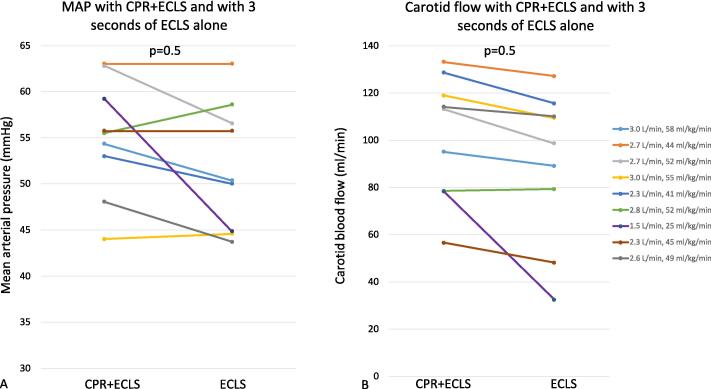
Mean arterial pressure and carotid flow averaged during 3 s before and after CPR cessation in the 9 animals in the study. A – mean arterial pressure in the 9 animals 3 s before (CPR + ECLS) compared with 3 s after (ECLS) cardiopulmonary resuscitation. The legend on the panel B shows the ECLS output expressed as Liters per minute and millilitres per kilogram per minute for each individual pig. Color codes and ECLS output are the same in panels A and B. B – Carotid flow in the 9 animals 3 s before (CPR + ECLS) compared with 3 s after (ECLS) cardiopulmonary resuscitation. MAP – mean arterial pressure, CPR – cardiopulmonary resuscitation, ECLS – extracorporeal life support.

Among the 16 pigs screened, 9 were eligible for inclusion in the study. Their weight was 54 (53–56) kg, the ECLS was initiated at 2.7 (2.3–2.8) L/min corresponding to 49 (44–52) ml/kg/min (Fig. 3), and the duration of the CPR + ECLS was 16 (7–33) seconds (Table 1). There was no significant bleeding recorded in any of the included animals during instrumentation or during the rest of the protocol.

**Table 1:** Characteristics of the animals during the experiment.

Parameter	Baseline	P*	CPR Alone	P*	CPR + ECLS	ECLS alone 3 s	P*	ECLS alone 6 s	P*	ECLS alone 30 s	P*	ECLS alone 5 min	P*	ECLS alone 10 min	P*
MAP	91 (86–100)	<0.001	37 (31–38)	0.001	56 (53–59)	50 (45–57)	0.50	52 (46–59)	0.61	61 (50–63)	0.70	57 (54–66)	0.44	54 (47–58)	0.73
CVP	0 (−5–5)	<0.001	23 (19–26)	0.06	18 (11–21)	9 (8–10)	0.02	10 (9–12)	0.08	10 (5–12)	0.04	9 (8–12)	0.063	8 (7–12)	0.034
ICP	23 (19–25)	0.80	24 (20–27)	0.90	24 (18–27)	21 (14–25)	0.52	23 (16–26)	0.80	21 (16–26)	0.61	24 (15–27)	0.83	24 (20–27)	0.92
Carotid Flow	263 (236–300)	<0.001	101 (78–117)	0.70	113 (78–119)	99 (79–110)	0.41	100 (81–110)	0.52	96 (60–122)	0.73	118 (101–130)	0.33	124 (110–141)	0.20

*Comparisons were made between the CPR + ECLS phase and the other phases of the study. ECLS – extracorporeal life support, CPR – cardiopulmonary resuscitation, NA – not applicable, MAP – mean arterial pressure, VF – ventricular fibrillation, CVP – central venous pressure, ICP – intracranial pressure.

### Hemodynamic parameters

Hemodynamic data in different phases of the study are represented in Table 1 and Fig. 3.

MAP did not decrease significantly between the CPR + ECLS, 56(53–59) mmHg *versus* ECLS alone, 50(45–57) mmHg at 3 s (Fig. 3), p = 0.50, 52(46–59) mmHg at 6 s(p = 0.61) and 61(50–63) mmHg at 30 s after cessation of CPR, p = 0.70 (Table 1, Fig. 3).

Carotid flow did not decrease significantly between the CPR + ECLS, 113(78–119) ml/min *versus* ECLS alone at 3 s, 99(79–110) mmHg, p = 0.41, 6 s 100(81–110) ml/min, p = 0.52 and 30 s 96(60–122) ml/min, p = 0.73 (Table 1, Fig. 3).

Similarly, no significant differences in these parameters were found at 5 min and 10 min (Table 1).

Central venous pressure decreased significantly from the CPR + ECLS to ECLS alone at 3 s, 30 s, and at 10 min (Table 1).

Intracranial pressure did not vary between different phases of the study (Table 1).

Among the 9 pigs, one was an outlier with a larger decrease in MAP from 59 to 45 mmHg, and carotid flow from 78.2 to 32.5 ml/min after 3 s of ECLS alone and 41.8 mmHg and 39.7 ml/min at 6 s respectively. The initial flow was lower than the rest of the pigs, 1.5 L/min, corresponding to 25 ml/kg/min (Fig. 3), the initial flow being at the discretion of the research team. After 25 s of ECLS alone, flow was increased to 2.7 L/min with an increase in MAP of 48 mmHg and carotid flow of 59.4 ml/min at 30 s. Flow was then increased to 3.38 L/min with an increase in MAP of 90 mmHg and 95 mmHg and carotid flow of 79 ml/min and 87 ml/min at 5 and 10 min, respectively.

Between 30 s and 5 min after CPR completion, vasopressors were initiated in 5 pigs and between 5 min and 10 min vasopressors were initiated in 4 pigs, and one pig underwent defibrillation with return of spontaneous circulation.

## Discussion

This study showed that stopping the CPR after initiating effective ECLS treatment is not associated with a significant decrease in the mean arterial blood pressure or carotid blood flow. This suggests that maintaining CPR in addition to full ECLS support does not improve key hemodynamic parameters. Noteworthy, the ECLS support was largely initiated at 2.7 L/min in the included pigs weighing 54 kg representing 50 ml/kg/min, a level of support that is recommended in humans in refractory cardiac arrest, 3-4L,^8^ this representing an ECLS flow of 40–53 ml/kg/min assuming an average person weighs approximately 75 kg. We did not find evidence of any differences in MAP or cerebral flow between CPR + ECLS and ECLS alone at 5 and 10 min after CPR completion. However, these results are interpreted with caution since vasopressors were initiated in 8 out the 9 pigs before the 10th minute and one pig was defibrillated and had return of spontaneous circulation. Therefore, stability of some of the circulatory parameters like systemic vascular resistance cannot be assumed between these timepoints.

These findings are relevant in the setting of effective ECLS with complete systemic support. However, augmentation with CPR may be prove more significant during ECLS with lower than recommended flow. We observed a large decrease in MAP and carotid flow after CPR cessation if ECLS was initiated at 1.5 L/min. Full ECLS support in humans is considered to be 90 ml/kg.^12^ In a pig model, 72 ml/kg is a sufficient level of support to ensure adequate perfusion.^13^ However, recent guidelines suggest that patients receiving ECLS for refractory cardiac arrest may be treated with 3–4 L/min flow,^8^ and 8 out 9 pigs in our study were treated accordingly. Central venous pressure decreased after CPR completion due to cessation of positive pressure generated by chest compression as shown in Fig. 1.

During the CPR + ECMO phase, the flow is pulsatile *versus* continuous flow with ECLS alone, and this may have an impact on microcirculation and organ perfusion.^14^ However, these effects may not be relevant at such short intervals as described in this study. We, therefore, did not focus on pulsatility.^14^.

Although thresholds of ECLS support may differ, whether a lower flow ECLS may benefit from additional CPR, may be explored in future models. Although not in use at present, earlier hemodynamic support in cardiac arrest may involve smaller bore cannulas which may be easier to insert, or shorter venous cannulas providing a lower flow in which case additional flow from CPR may improve overall perfusion.

CPR in addition to circulatory assisting devices may be also useful for strategies focusing specifically on regional perfusion of the brain as suggested by Bellomo *et al,* who showed improved brain oxygenation using carotid cannulation and direct perfusion of the brain, while the systemic organ perfusion was supplied by direct cardiac compressions.^7^.

In our study we chose to evaluate parameters at short intervals because we assumed stability of the physiologic parameters like vascular repletion, systemic circulatory resistance, and stability of the ECLS flow. Refractory cardiac arrest is a highly unstable condition requiring many interventions such as vascular repletion, vasopressors, adjustment of the ECLS flow to maintain mean arterial pressure at 65 mmHg. These interventions performed on longer time intervals modify the circulatory parameters making the interpretation of the results difficult. We therefore focused on comparing the parameters of interest at the end of the treatment with CPR + ECLS, with the parameters at 3, 6 and 30 s, 5 and 10 min of ECLS alone. In the interpretation of the data, we assumed that the main circulatory parameters remained constant, and that changes in pressures and carotid flow were attributed to the cessation of CPR at 3, 6, and 30 s timepoints. At the 5th and 10th minute of ECLS alone, MAP and carotid flow were not significantly different from the CPR + ECLS, but these findings are confounded by initiation of vasopressors in 8 animals and defibrillation with return of spontaneous circulation in another animal.

Our study has several limitations, one being the relatively reduced number of pigs, although this analysis making every animal its own control reduces inter-individual variability and the need for large numbers of animals. Another limitation is the non-standardized duration of overlap between CPR and ECLS which in some pigs was as short as 5 s, and the fact that the initial ECLS flow was not initiated at the same level in all pigs, and several levels of ECLS flow were not evaluated. This is inherent to the nature of our study which is an ancillary observational analysis of hemodynamic data, but may be performed in future studies. Our study did not evaluate neurological outcome or survival of the animals. The study was not designed for this purpose, and only focused on key physiological parameters such as carotid flow and mean arterial pressure.

The common carotid instead of internal carotid flow was measured due to the porcine anatomy. The internal carotid is a small intracranial branch arising from the arterial meshwork rete mirabilis at the base of the skull. As a result, the internal carotid is inaccessible by routine surgical technique for anatomical reasons.^15^.

Our study did not measure tissue oxygen saturation of the brain using techniques like near-infrared spectroscopy due to difficulties in measurement in adult pigs. This technique is more appropriate in piglets than in adults pigs, although recent versions like hyperspectral time-resolved near-infrared spectroscopy may become feasible and reliable in the future.^16^ Transcranial doppler was not performed as well due to pig skull and vascular anatomy despite being an effective clinical tool in humans. It is unreliable and not reproductible in adult pigs, especially under dynamic conditions of cardiopulmonary resuscitation.

In conclusion, our study did not find a significant decrease in mean arterial pressure or carotid flow when CPR performed in addition to effective ECLS was stopped, and animals remained on ECLS alone. Future studies may explore the additive effect of CPR in the setting of submaximal ECLS support, in which CPR may augment key physiological parameters like mean arterial pressure and carotid flow.

## CRediT authorship contribution statement

**Sergey Gurevich:** Writing – original draft, Investigation, Conceptualization. **Rajat Kalra:** Writing – review & editing, Validation, Methodology. **Marinos Kosmopoulos:** Writing – review & editing, Validation. **Alexandra M Marquez:** Writing – review & editing, Validation. **Deborah Jaeger:** Writing – review & editing, Validation, Investigation. **Mitchell Bemenderfer:** Writing – original draft, Validation. **Danielle Burroughs:** Writing – review & editing, Validation. **Jason A Bartos:** Writing – review & editing, Validation, Conceptualization. **Demetris Yannopoulos:** Writing – review & editing, Validation, Supervision, Conceptualization. **Sebastian Voicu:** Writing – review & editing, Validation, Supervision, Conceptualization.

## Declaration of competing interest

The authors declare that they have no known competing financial interests or personal relationships that could have appeared to influence the work reported in this paper.
